# Proteasomes and Ubiquitin C-Terminal Hydrolase L1 as Biomarkers of Tissue Damage and Inflammatory Response to Different Types of Injury—A Short Review

**DOI:** 10.3390/life15030413

**Published:** 2025-03-06

**Authors:** Marzena Tylicka, Ewa Matuszczak, Joanna Kamińska, Beata Modzelewska, Olga Martyna Koper-Lenkiewicz

**Affiliations:** 1 Department of Biophysics, Medical University of Bialystok, Mickiewicza 2a, 15-222 Bialystok, Poland; beata.modzelewska@umb.edu.pl; 2 Department of Pediatric Surgery, Medical University of Bialystok, Waszyngtona 17, 15-274 Bialystok, Poland; ewa.matuszczak@umb.edu.pl; 3 Department of Clinical Laboratory Diagnostics, Medical University of Bialystok, Waszyngtona 15A, 15-269 Bialystok, Poland; joanna.kaminska@umb.edu.pl (J.K.); olga.koper-lenkiewicz@umb.edu.pl (O.M.K.-L.)

**Keywords:** proteasome, ubiquitin-proteasome system, UCHL1, trauma, injury, head trauma, burns, thermal injury, ischemia-reperfusion, abdominal trauma

## Abstract

The proteasomal system of protein degradation is crucial for various cellular processes, including transduction of signals and differentiation of cells. Proteasome activity rises after various traumatic stressors such as hyperoxia, radiation, or oxidative damage. Removal of damaged proteins is essential to provide the necessary conditions for cell repair. Several studies report the activation of the proteasomal degradation system after thermal injury, CNS injury, abdominal trauma, ischemia-reperfusion injury, and possible clinical implications of the use of proteasome inhibitors. It is important to highlight the distinct and crucial roles of UCHL1, 26S, and 20S proteasome subunits as biomarkers. UCHL1 appears to be particularly relevant for identifying brain and neuronal damage and in advancing the diagnosis and prognosis of traumatic brain injury (TBI) and other neurological conditions. Meanwhile, the 26S and 20S proteasomes may serve as markers for peripheral tissue damage. This differentiation enhances our understanding and ability to target specific types of tissue damage in clinical settings.

## 1. Proteasomes 20S and 26S-Structure, Role and Function

**Figure 1:**
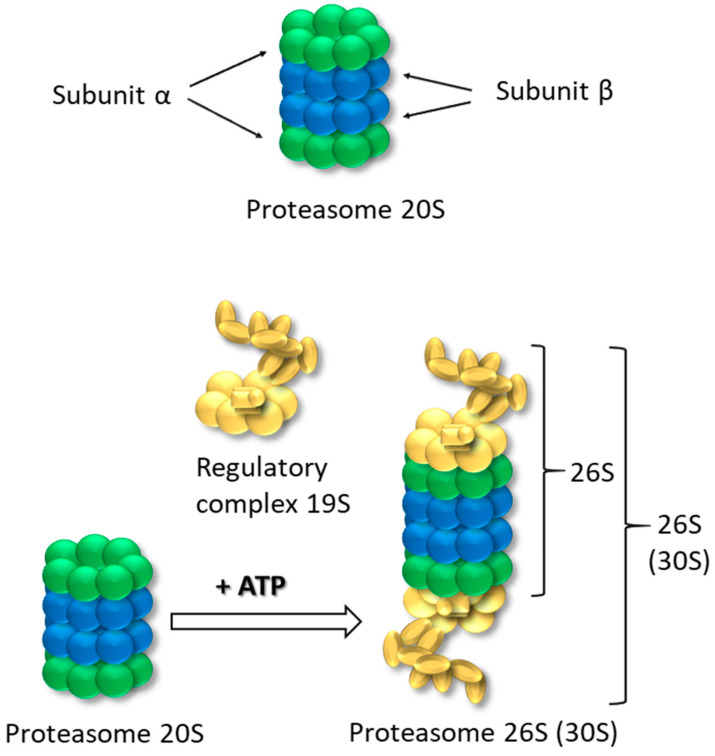
Structure of 20S and 26S proteasome.

Proteasomes are multicatalytic enzymes responsible for the degradation of proteins [1,2]. They also regulate many proteins influencing the inflammatory response, growth, and differentiation of cells [1,2]. 26S proteasomes are composed of two separately stable and functionally distinct sub-complexes: the core protease 20S, which is responsible for the peptidase activities, and regulatory particle 19S, which captures and prepares protein substrates for breaking down [3]. The central and proteolytic part of the 26S proteasome (the core protease 20S) is built of four rings, with seven subunits in each ring (Figure 1) [1,2]. The outer ring subunits are called α subunits and the inner ring subunits are called β subunits [4,5]. The α subunits interact with regulatory proteins and the β units have a catalytic function [4,5,6,7]. In eukaryotic proteasomes, only three β-subunits are catalytically active [7]. β-rings contain six catalytic sites responsible for peptide bond cleavage, which are provided by the β_1_, β_2_, and β_5_ subunits. The β_1_, β_2_, and β_5_ sites provide trypsin-like, chymotrypsin-like, and caspase-like cleavage properties, respectively [3]. β_1_ and β_2_ active sites cleave acidic and basic residues, while the β_5_ active site cleaves hydrophobic residues [7]. The α-subunit rings are located on the top of β-subunit rings, and their primary function is to regulate the entry of proteins into the central proteolytic chamber. Proteasome gate opening is triggered by interaction with various proteasome regulators, such as the regulatory particle 19S (PA700), or the activators PA28ab, PA28g, PA200, PI31, and Cdc48. These regulatory complexes control the opening and closing of the gated pore [3,7]. The primary regulator of the 20S core protease is the 19S regulatory particle, which binds to one or both ends of the proteasome in an ATP-dependent manner, forming either the singly capped 26S proteasome or the doubly capped 30S proteasome, respectively. The 26S proteasome is present in both the cytoplasm and nucleus of all eukaryotic cells, whereas the 30S proteasome has been identified in archaeal and bacterial cells. However, the term “26S proteasome” is commonly used to refer to both forms. The 19S regulatory particle plays a crucial role in substrate processing by recognizing ubiquitylated proteins, facilitating their unfolding, opening the α-ring pore, translocating substrates into the proteolytic core, and recycling ubiquitin moieties before substrate degradation [8].

## 2. 20S Proteasome Ubiquitin-Independent Protein Degradation

Two main types of proteasomes coexist in most cells, and half of them are free 20S proteasome complexes. Degradation by the 20S proteasomes does not require the presence of the regulatory particles 19S and proteins ubiquitin tagging. These proteasome complexes are responsible for the degradation of proteins that contain unstructured regions due to oxidation, mutation, or aging, as well as naturally, intrinsically unfolded proteins [9]. The primary requirement for proteins that are subjected to proteasomal degradation in a ubiquitin-independent pathway is the presence of unstructured regions in proteins. This kind of proteasomal degradation mechanism is mediated only by the 20S proteasome complex, without the assistance of regulatory complex 19S. Substrates intended for degradation in a ubiquitin-independent manner may be native proteins with large unstructured segments with more than 30 amino acids in length or proteins with entirely disordered sequences. The second group of these substrates plays essential roles in cell cycle progression, cellular growth control, and oncogenesis. Therefore, the role of proteasome 20S in their degradation is significant because their levels must be tightly controlled. Alterations in their amount may lead to the development of various diseases [9].

Several studies indicated the significance of the ubiquitin-independent 20S proteasome degradation route. The majority of proteasomes in mammalian cells are represented by proteasome 20S, while only about 20–30% are identified as 26S proteasomes [9]. Moreover, it was shown that in mammalian cell extracts, more than 20% of the cellular proteins undergo 20S proteasome degradation. In contrast to 26S proteasomes, 20S proteasomes are more resistant to oxidative stress and maintain their activity under conditions in which protein damage occurs [9].

Previous studies demonstrated that active Proteasomes 20S are identified in various extracellular compartments such as plasma, serum cerebrospinal fluid, and extracellular bronchoalveolar lavage fluids; therefore, they are called “circulating” or extracellular proteasomes. Their level is elevated under stress conditions such as injury and various diseases and in different types of cancers. Turker et al. suggested that 20S proteasome cores are found in the nervous system, which is crucial for neuronal activity-dependent signaling [10]. Elevated proteasome 20S levels were found in human serum in response to aggressive hematologic malignancies, inflammatory liver disorders, melanoma, sepsis, surgeries, burns, or inhalation injuries. Ben Nissan et al. indicated that decreased proteasome 20S levels are found in patients in early stages of chronic lymphatic leukemia and multiple myeloma non-Hodgkin’s lymphoma, but the increase is observed in more aggressive stages [11].

20S proteasome is circulating in plasma, and its increased activity was found in patients with trauma, neoplastic tumors, autoimmune diseases, or critical illness. The proteasome concentration reflects the degree of cell damage, regardless of the cause [4,9,12].

## 3. 26S Proteasome Ubiquitin-Dependent Protein Degradation

**Figure 2:**
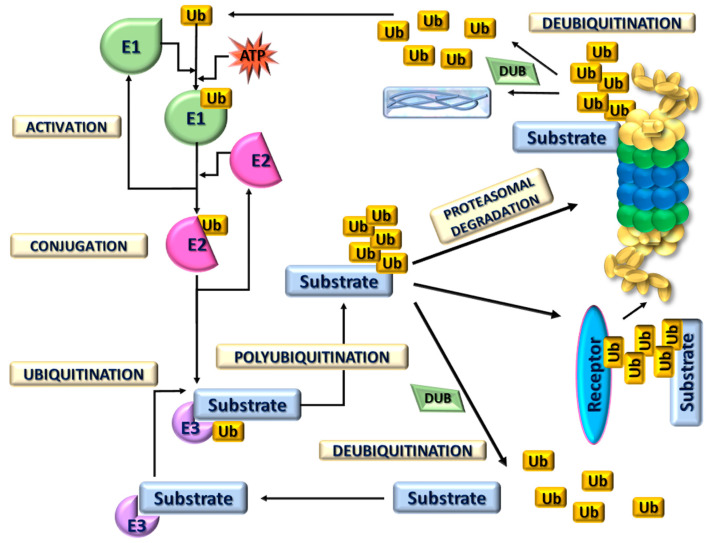
Ubiquitin-proteasome mediated pathway of proteolysis. A ubiquitin (Ub) molecule is first activated via a ubiquitin-activating enzyme (E1) through an ATP-dependent reaction (Activation). Then, the activated Ub is transferred to a ubiquitin-conjugating enzyme (E2) (Conjugation) and the target substrate by an E3 ubiquitin ligase (Ubiquitination). This ubiquitination process can be reversed by deubiquitinating enzymes (DUBs) (Deubiquitination). Following several rounds of ubiquitination (Polyubiquitination), the polyubiquitinated substrates are either delivered to the 26S proteasome by Ub receptors or directly recognized by the 26S proteasome complex (Proteasomal degradation). The polyubiquitin molecules are then disassembled by DUBs to small peptides and amino acids and recycled for new rounds of ubiquitination.

Proteasome 20S complex may also exist as a core of proteasome 26S complexes associated with ubiquitin-dependent degradation. Proteins destined for breakdown are attached to a ubiquitin molecule, binding to other ubiquitin molecules to form the “tail” of the protein. Attachment of ubiquitin is a crucial step that contributes to numerous biological processes such as embryonic development, the cell cycle, growth control, and prevention of neurodegeneration. Conjugation of ubiquitin to a substrate requires three different enzymes, E1, E2, and E3, that create highly dynamic structures. The first is a ubiquitin-activating enzyme that is responsible for ATP-dependent activation of the C-terminus of ubiquitin and for forming a covalent bond with Ub. After this step, ubiquitin is transferred to the ubiquitin-conjugating enzyme or E2. Finally, the third enzyme catalyzes the transfer of ubiquitin to a protein substrate (Figure 2) [13]. These ubiquitin-activating enzymes facilitate the formation of an isopeptide bond between the C-terminal glycine (G76) of ubiquitin and the ε-amino group of a lysine residue on the substrate protein [14,15]. The human genome encodes 2 E1s, approximately 38 E2s, and over 600 E3s. E3 ligases have the largest number of encoding genes among the three ubiquitination enzymes, highlighting their role in precise substrate recognition [14]. Based on structure and function, E3 ligases are classified into four types: HECT (homologous to the E6AP carboxyl terminus), U-box, RING (really interesting new gene) finger, and RBR (RING-IBR-RING) types. Notably, these E3 ligase types exhibit low sequence homology and distinct structural differences [16]. It should be highlighted that E3 ligases have significant therapeutic potential, particularly in targeted protein degradation through PROTACs (proteolysis-targeting chimeras) for treating cancers and neurodegenerative diseases [14]. Their regulation of key pathways, such as p53 and NF-κB, makes them valuable targets for cancer therapy and immunomodulation. Enhancing E3 ligase activity could help clear toxic protein aggregates in neurodegenerative disorders like Alzheimer’s and Parkinson’s. Additionally, E3 ligases are promising targets for antiviral strategies, gene therapy, and drug discovery [14,15,16].

Only proteins tagged by the polyubiquitin chains are degraded by the proteasomes 26S. The ubiquitin-dependent 26S proteasomal degradation process refers to damaged and short-lived regulatory proteins tagged by the ubiquitin chain [9]. The ubiquitin-proteasome system (UPS) is responsible for the degradation of most intracellular proteins, such as many proteins critical to cellular regulatory pathways, including cell-cycle progression, DNA damage repair, and antigen presentation [7].

There is evidence that important regulators of the ubiquitin-proteasome system are deubiquitinating enzymes, which may be responsible for proof-reading ubiquitin-protein conjugates, removing ubiquitin, and keeping the 26S proteasome free of inhibitory ubiquitin chains [13]. The human genome encodes approximately 100 distinct DUBs, which are classified into 7 subfamilies. Six of these subfamilies are cysteine proteases, including ubiquitin-specific proteases (USPs), ovarian tumor-like proteases (OTUs), ubiquitin C-terminal hydrolases (UCHs), Machado–Joseph disease protein domain proteases (MJDs), MIU-containing novel DUB family (MINDY) proteases, and zinc finger ubiquitin-specific protease (ZUFSP/ZUP1). The seventh subfamily, Jab1/Mov34/Mpr1 (JAMM) proteases, consists of metalloproteinases [17,18]. The UCH family encompasses four members, UCHL1, UCH-L3, UCH37, and BAP1 (BRCA1-associated protein-1) [17,18]. UCHs feature an N-terminal C12 peptidase domain with a knotted peptide backbone, a C-terminal extension, and an unstructured loop that controls substrate access to the catalytic site [19]. Ubiquitin C-terminal hydrolase-L1 (UCHL1) exhibits both deubiquitinase and ubiquitin (Ub) ligase activities, playing a key role in Ub stabilization [17]. The UCHL1 gene, also known as the PARK5 gene, is located on chromosome 4p14 [17]. The UCHL1 peptide consists of 223 amino acids with a molecular weight of approximately 24.8 kDa. Its structure closely resembles that of UCH-L3. Each UCHL1 monomer features two lobes: one containing five α-helices and the other comprising two α-helices and six β-sheets. Together, these α-helices and β-sheets form the characteristic α-β-α structure of UCHL1 [17].

The ubiquitin C-terminal hydrolase L1 (UCHL1) is a deubiquitinating enzyme with hydrolase and ligase activity. As a hydrolase, UCHL1 takes part in the recycling and removal of ubiquitin from proteins undergoing degradation. As a ligase, UCHL1 binds molecules of ubiquitin which tag proteins intended for degradation [20,21].

UCHL1 is mainly expressed in brain and neuroendocrine tissue, and “accounts for 1–2% of all brain soluble proteins” [21]. In vitro inhibition of UCHL1 results in a 50% reduction of free ubiquitin [22]. It also acts as a slow stabilizer of ubiquitin, providing it for cellular processes. The ubiquitin-proteasome system can also reverse the ubiquitination, disassembling the poly-ubiquitin chain and preventing protein degradation [20,21,22]. The state of a protein is regulated by both attachment and cleavage of ubiquitin, changing its lifetime or biochemical function. UCHL1 can also “stabilize cellular proteins such as β-catenin and activate kinases such as CDK4” [20,21]. The function of UCHL1 is different depending on the environment. UCHL1 exerts its action by influencing the concentration of free proteins and peptides, such as ubiquitin and glutathione, and regulating the cell cycle [20,21,22]. In addition, “a functional NF-κB response element has been identified in the UCHL1 promoter region” [21]. NF-kB (nuclear factor kappa-light-chain enhancer of activated B cells) is a protein complex controlling the transcription of DNA, cytokine production, and cell survival [23]. NF-kB exists in the cytoplasm in an inactive complex bound to IkB. Most agents that activate NF-kB do so through proteasome-mediated degradation of IkB [23]. Inflammation likely impairs the function of neurons through the interaction of NF-κB and UCHL1 [20]. Lower levels of UCHL1 were observed in Alzheimer’s disease and amyotrophic lateral sclerosis [22,24]. Without UCHL1, cortical spinal motor neurons undergo degeneration by progressive cytoarchitectonic changes [25]. During neurodegenerative diseases, aggregates of abnormal proteins accumulate within the neurons [22,23,24,25,26]. In the UCHL1 promoter region, a response element for the Nuclear Factor NF-κB was found, and NF-κB suppresses gene transcription of UCHL1 [20]. According to Wang et al., activation of NF-κB by lipopolysaccharide and TNFα suppressed the transcription of the UCHL1 gene [20]. As NF-κB is an important signaling module in the inflammatory response, a study by Wang et al. suggests that inflammation can compromise neuronal function by NF-κB and UCHL1 interaction, explaining the role of inflammation in the pathogenesis of Alzheimer’s and Parkinson’s diseases [20,22,23,24,25,26]. Research on blood-based biomarkers of traumatic brain injury (TBI) has accelerated significantly over the past decade, including UCHL1 (Table 1). Serum concentration of UCHL1 rises within the first 6–24 h after TBI and correlates with injury severity and clinical outcomes, including GCS score at admission and computed tomography (CT) lesions [27]. In patients who tested positive on CT scans, serum levels of UCHL1 measured 6–12 h post-injury were higher in those with unfavorable neurological outcomes [28]. In the cohort study conducted by Papa et al., the levels of glial fibrillary acidic protein (GFAP), UCHL1, and microtubule-associated protein 2 (MAP-2) measured within 30 and 60 min of injury were found to be significantly associated with traumatic intracranial lesions and diffuse injury severity on CT scan, as well as 24-h neurological severity index (NSI) and 7-day comprehensive impact of event outcome (CIEO) [29]. Lewis et al. conducted a study to assess the ability of GFAP, UCHL1, and S100B to stratify emergency department patients with biomarker levels measured within 6 h of injury. They found that UCHL1 levels were significantly higher in patients with complicated mild TBI (mTBI) compared to those with uncomplicated mTBI and in patients with mTBI compared to those without a traumatic brain injury. UCHL1 showed a sensitivity of 95% in distinguishing between no TBI and mTBI [30].

The United States Food and Drug Administration (FDA) approved the use of blood-based biomarkers, GFAP and UCHL1, in 2021 for predicting intracranial lesions on head CT scans in patients with mTBI. It was found that UCHL1 and GFAP levels had a sensitivity of 97% and a specificity of 19% for predicting a positive CT scan result in patients with mTBI, with a negative predictive value of 95% and a positive predictive value of 27%. Combining biomarker analysis with loss of consciousness and time to sample collection increased the specificity to 46% [31]. Currently, validated and approved in vitro diagnostic (IVD) tests are available for routine use in the diagnosis of TBI using biomarkers such as GFAP and UCHL1, both in laboratory settings and at the point of care (i-STAT Alinity, TBI Test Alinity, Abbott Diagnostics, Princeton, NJ, USA) and VIDAS^®^ TBI, GFAP, UCH-L1, (BioMerieux Marcy-l’Étoile, France) [31,32,33].

Deregulation of the ubiquitin-proteasome system contributes to the pathogenesis of several human diseases, such as cancers and neurodegenerative and myodegenerative diseases [4,5,6,7].

Recent studies showed that proteasomes in the nervous system generate peptides with important cellular functions. Therefore, the dysregulation of protein degradation machinery contributes to the initiation or progression of neurodegenerative diseases, such as Alzheimer’s disease, Parkinson’s disease, and Huntington’s disease. Proteasome ubiquitin-dependent protein degradation plays a crucial role in various aspects of neuronal development, including neurogenesis, differentiation, and synapse formation. Its proper function is therefore essential for a healthy nervous system [10]. Circulating proteasomes have been found in blood samples from patients with systemic lupus erythematosus, multiple sclerosis, and rheumatoid arthritis [34]. It is interesting to record that in neurological diseases, E3 ubiquitin ligases have been discovered to be mutated or dysregulated. These ubiquitin-conjugating enzymes have been found in patients with autism or autism spectrum disorders [10].

The authors also demonstrated that aberrations in the ubiquitination pathway have been associated with many cancer types. Ubiquitination plays numerous functions, such as modulating protein activity, activating enzymes, facilitating DNA repair, and regulating chromatin structure. This multifaceted involvement indicates a significant role for this process in tumorogenesis. Dysregulation of proteasomal degradation, both its acceleration and reduction, is implicated in numerous diseases, including cancer. It is also postulated that the inhibition of the chymotrypsin-like activity of tumor-cell is postulated to be a potent stimulator of apoptosis. Therefore, the proteasome might be a target for anticancer therapy [35]. The reverse process of ubiquitination is equally important and is collectively performed by deubiquitin enzymes (DUBs), which have been shown to regulate DNA damage response. DUBs are crucial regulators of chromatin remodeling, DNA damage repair, and histone modification [36].

Numerous studies have revealed that proteasome has a detrimental effect on the replication of SARS-Coronavirus-2 and other viruses. Tola suggested that proteasome inhibition may have an influence on viruses. Ubiquitination may play a crucial role in the early stages of virus-host entrance. The processes of ubiquitination and proteasome-dependent destruction may constitute strategies to restrict virus entry. Well-known characteristics of severe COVID-19 patients are hypoxemia, a decrease in T cells, and an inflammatory storm situation. Conducting investigations on COVID patients, researchers demonstrated that hypoxia might increase the mRNA expression of proteasome subunits, which may be related to lymphocyte cell death [37].

Based on data and the findings of studies on eye diseases, it appears reasonable to suggest that ubiquitin can play a role in eye diseases such as cataracts, glaucoma, keratopathy, retinopathy, and eye tumors. The occurrence of eye diseases is affected by many factors, such as genetic and nongenetic factors, in which the role of ubiquitin is significant. Ma et al. recently demonstrated that reactive oxygen species production and apoptosis of the human lens caused the cataract. Moreover, E3 ubiquitin ligases have an impact on cataract development. They pointed out that the mechanism of protein ubiquitination, which affects eye disease, mainly focuses on the proteasome, and the ubiquitination of key proteins inhibits and degrades their functional activity, inducing inflammation and apoptosis [38].

Recent evidence indicated the role of ubiquitin-proteasome system dysfunction in the development of cardiovascular disease. Performed research focusing on cardiovascular disease pathophysiology demonstrated the significance of UPS-mediated oxidative stress, which plays a major role in this disease. Cardiac proteins are in dynamic states of degradation and synthesis and the ubiquitin-proteasome system regulates their turnover and further plays a role in the cellular response to oxidative stress. Qiu et al. observed increased levels of oxidative stress and proteasomes in patients with dilated cardiomyopathy. The UPS is also expected to play a pathophysiological role in heart failure [39].

Some authors link the ubiquitin-proteasome system with inflammatory processes. According to Matuszczak et al., in the plasma of children with acute appendicitis, the levels of UCHL1 are increased [40]. Furthermore, Zhang et al. showed that nephropathy related to type IV collagen could be treated with inhibitors of the ubiquitin-proteasome system [41]. There are observations that UCHL1 may also modulate vascular remodeling [42]. UCHL1 attenuated the activity of NF-kβ (nuclear factor kβ) induced by TNF-α (tumor necrosis factor) and increased the expression of eNOS, reducing ischemic vascular complications in atherosclerosis [42]. Endothelial UCHL1 may also influence intraocular neovascularization [43]. Except for CNS, UCHL1 is also expressed in the testis and ovaries, and its increased expression was reported in different types of cancer (e.g., brain, lung, breast, kidney, colon, prostate, pancreas). The role of UCHL1 in different types of cancer remains controversial. Some studies have identified UCHL1 as either an oncogene or a tumor suppressor, depending on the context [44,45,46,47,48,49]. Yu et al. demonstrated the tumor-suppressing activity of UCHL1 in gastrointestinal cancers [47]. Conversely, other researchers have characterized UCHL1 as an oncogene in cancers such as non-small-cell lung cancer, lymphoma, prostate cancer, colorectal cancer, melanoma, and osteosarcoma [50,51,52,53,54]. Additionally, Wu et al. proposed that UCHL1 may serve as a biomarker and therapeutic target in metastasis in gastric cancer [52].

Mansoor et al. postulated the ATP-ubiquitin-dependent proteolytic pathway in muscle wasting following injury [55]. The ubiquitin-proteasome system in skeletal muscles labels and then degrades proteins for destruction [55]. Rodent and patient studies have confirmed that the ubiquitin and proteasome protein degradation pathway significantly contributes to skeletal muscle wasting [56,57]. Seiffert et al. reported elevated proteasome activity in muscle after trauma, but Farges et al. did not find changes in the activity of the proteasome after muscle injury [56,57].

## 4. Proteasomal System of Protein Degradation in Response to Different Types of Injury

Proteostasis is a mechanism of cellular homeostasis ensuring the sensitive balance between the synthesis, folding, trafficking, and degradation of proteins. Particularly important is protein trafficking, which sorts all proteins into their distinct destinations to ensure their correct function. Ageing and many pathological conditions such as infection, inflammation, or oxidative stress, highly changed proteostasis, which results in protein damage or misfolding. The ubiquitin-proteasome system is an essential protein quality control system that opposes disrupted proteostasis and prevents the development of proteinopathies [58] (Table 1).

The activity of the ubiquitin-dependent or independent proteasome system rises after various traumatic stressors. Therefore, it is necessary to emphasize the role of proteasomal degradation systems in different types of injury and the possible clinical implications of using proteasome inhibitors.

### 4.1. Central Nervous System Injury

After head trauma, patients are commonly admitted to Emergency Departments. We are still in need of new markers for the severity of head injuries.

The ubiquitin-proteasome system regulates cell death in ischemic neurons [59,60]. A study by Szabo et al. found that “brain injury increases the level of protein oxidation in the damaged cortex”. According to Urso et al., “the activity of the ubiquitin and proteasome…increase significantly two and five days after injury of the spinal cord” [60]. Changes in the activity of the proteasome were also observed after head trauma in the pediatric population [61]. Elevated activity of the circulating proteasome was reported in children with mild head trauma, correlating with the severity of concussion symptoms. A decreasing trend in plasma proteasome activity was observed two days after head trauma, which was consistent with the resolution of concussion symptoms [61].

Currently, many researchers agree that UCHL1 is a well-accepted serum biomarker for severe traumatic brain injury (TBI) [62]. UCHL1 concentration rises significantly in peripheral blood shortly after injury and correlates with long-term clinical outcomes [62,63,64]. Jones et al. postulate that UCHL1, serving as a biomarker of the severity of brain trauma, could reduce unnecessary radiation exposure [64]. Some authors suggested that UCHL1 may be a more effective predictor of CNS trauma severity compared to calcium-binding growth-regulating secretory protein S100B or myelin basic protein (MBP) [65]. In addition, elevated UCH-L1 levels were connected with CNS injury while CT was still negative [65]. An example is a study by Mondello et al., who demonstrated that higher levels of UCHL1 help to recognize children with the possibility of poor outcomes, granting more specific treatment [66]. Moreover, in newborns with hypoxic-ischemic encephalopathy, high levels of UCHL1 were associated with increased severity of the brain injury detected by MRI [67].

### 4.2. Thermal Injury

Thermal injury causes an integrated metabolic response that impairs the function of organs. It triggers progressive increases in energy expenditure, a significant decrease in total protein in the body, and organ dysfunction with the risk of developing multiple organ failure and sepsis [68]. The main mechanisms contributing to skeletal muscle wasting thermal injury involve the activation of the ubiquitin-proteasome pathway and increased degradation of calcium-dependent and lysosomal proteins [69,70,71]. Matuszczak et al. studied the circulating proteasomes in pediatric patients with burns [69,70]. The circulating proteasome activity was elevated until the 7th day after severe burns, by the 5th day after moderate burns, and by the 3rd day after minor burns; it correlated with the degree of burn but did not correlate with gender or age [69]. Taken together, the peak activity of the 20S proteasome after burn injury, as well as its gradual decline, suggest that this proteasome may serve as a marker of tissue damage [69,70].

The changes in the plasma levels of UCHL1 were also observed after burns. Levels of UCHL1 peaked 12–16 h after thermal injury and then slowly decreased. UCHL1 concentration was proportional to the degree of thermal injury, reflecting the metabolic response of the organism [71].

### 4.3. Abdominal Injury

Abdominal injury is one of the most common injuries among children. Patients exhibit, among other things, enhanced rates of whole protein body breakdown. The degradation of damaged proteins is essential to provide the necessary conditions for cellular repair and plasticity Matuszczak et al. compared changes in the activity of the 20S proteasome in the plasma of pediatric patients after burns and head and abdominal trauma. The authors showed an increase in circulating proteasome activity in the plasma of children with abdominal injury. The circulating proteasome activity increased significantly 12–16 h after trauma and decreased 44–48 h after the injury. These results demonstrated that molecular changes affecting protein metabolism occur 12–16 h after injury and may reflect cellular damage [72].

### 4.4. Tissue Injury

Severe trauma induces an integrated metabolic response and consequently impaired function of virtually all organs and tissues of the body. Evidence suggests an important role for the ubiquitin-proteasome pathway in regulating intermediary metabolism and cell function. The turnover of defective proteins provides new material for cell repair. Activation of the ubiquitin-proteasome system was found after different injuring factors e.g., radiation or oxidative stress [73]. It is suggested that the ATP-ubiquitin-dependent proteolytic pathway is involved in muscle wasting due to injury. The role of the proteasome pathway in skeletal muscle is to tag and degrade proteins intended for destruction. Performed experiments showed proteasome activation in mechanically injured human skeletal muscle [72].

Patel et al. confirmed that proteasome peptidase activities are significantly elevated in direct mechanically injured human skeletal muscle. These findings demonstrated the tissue-specific roles of the ubiquitin-proteasome system in health and disease states. Based on the evidence regarding the major biological role of UPS in eukaryotic cells, they hypothesized that the ubiquitin-proteasome system is also involved in the whole-body response to severe trauma [73].

### 4.5. Inflammation-Associated Injury

Liu et al. found that UCHL1 is elevated in podocytes in different types of glomerulonephritis [74]. Zhang et al. claim that “NF-κB upregulates UCHL1 in diseased podocytes in glomerulonephritis” [75]. Furthermore, UCHL1–deficient mice exhibited an exacerbated course of disease with increased tubulointerstitial and glomerular damage, acute renal failure, and death [76].

UCHL1 may also serve as an indicator of pancreatic beta cell damage in vitro [77]. Recent research has demonstrated that UCHL1 inhibition suppresses hepatic stellate cell proliferation both in vitro and in vivo, preventing disease progression even in the setting of ongoing liver damage [78].

### 4.6. Ischemia-Reperfusion Injury

In the literature, there is a growing number of surveys connecting proteasome and ischemia-reperfusion injury. The proteasome activity was detected following transient cerebral ischemia and ischemia-reperfusion injury of the heart in animal models [79,80,81,82,83]. During ischemia-reperfusion, the proteasome degrades IκB proteins, known as regulators of NF-κB activity [23,83]. During oxidative stress, “NFκB is translocated into the nucleus and induces the production of proinflammatory cytokines (IL-6, LI-1, TNFα, COX2, IFNγ)” [23,82]. Because NF-kB has a major role in cell damage after reperfusion, Bao et al. used PR-39 and PR-11 proteasome inhibitors and achieved the reduction of infarct size in rat hearts after ischemia-reperfusion injury [84]. Similar results were reported by Pye et al.; according to their observations, the proteasome inhibitors decreased the neutrophil infiltration and reduced the inflammatory response after reperfusion [85].

In the 1960s, Brown et al. observed that the activity of 26 proteasomes in cold livers preserved for transplantation is lessened [79]. Decreased proteasome activity during ischemia causes the accumulation of abnormal proteins that are toxic to cells [79]. According to Oliva, all organs undergoing the ischemia-reperfusion injury share the same types of injury, such as impairment of microvascular function, inflammation, and cell death. One study demonstrated that use of proteasome inhibitors may decrease ischemia reperfusion injury by blocking or reducing many common factors involved in organ injury, such as the oxidative stress, the decrease of the 26S and 20S proteasome activity, the activity of NFκB, the inflammatory cell filtration, and the inflammatory response [83].

The table below summarizes the key findings concerning different types of injury.

**Table 1:** Proteasomal system of protein degradation in response to different types of injury.

Type of Injury	Key Findings
Central nervous system injury	- Spinal cord injury (SCI) induces early upregulation of genes in the ubiquitin-proteasome pathway (UPP), metallothioneins, and protease inhibition. Increased protein levels of PSMD11 (26S proteasome non-ATPase regulatory subunit 11) and metallothioneins, especially at the cell periphery, suggest targeted protein degradation, emphasizing the UPP’s role in muscle remodeling and stress response after SCI [60]. - Brain injury disrupts proteasome function, increasing oxidative stress and reducing synaptic plasticity. Exercise mitigates these effects by normalizing proteasome activity, reducing oxidative damage, and preserving synapsin I levels. This suggests that exercise supports protein homeostasis and synaptic plasticity after brain trauma by modulating proteasome-related pathways [59]. - Plasma c-proteasome activity significantly increases 2–6 h and 12–16 h after mild traumatic brain injury in children, peaking with concussion symptoms and decreasing by the second day as symptoms resolve [61]. - UCHL1 and GFAP are potential serum biomarkers for brain damage in neonates with hypoxic-ischemic encephalopathy (HIE). UCHL1 levels correlate with cortical injury and developmental outcomes, while changes in GFAP predict motor development and injury to basal ganglia and white matter. These biomarkers warrant further investigation for their role in assessing brain injury and neurodevelopment in HIE infants [67]. - GFAP and UCHL1 are promising biomarkers for pediatric traumatic brain injury (TBI), with serum levels correlating with injury severity and poor outcomes. UCHL1, in particular, is effective in detecting acute intracranial lesions and undetected microstructural injuries, outperforming S100B and MBP in predicting outcomes. These biomarkers hold potential for improving TBI diagnosis and prognosis in children [66]. - A panel of protein biomarkers (S100B, NSE, GFAP, UCHL1, tau, NF-L) effectively correlates with TBI severity and outcome. UCHL1 demonstrated the best discriminatory power for unfavorable outcomes, while GFAP and NF-L provided additional independent predictive value. These biomarkers, with varying cellular origins and temporal patterns, enhance the prediction of TBI outcomes [63].
Thermal injury	- Circulating 20S proteasome activity increases after burns in children and negatively correlates with plasma total protein levels, suggesting its potential as a biomarker for tissue damage in pediatric burns [70]. - Circulating 20S proteasome levels rise after burn injury in children and gradually normalize during wound healing. The increase correlates with burn severity but is independent of age and sex [69]. - UCHL1 concentration increases within 2–16 h after thermal injury and gradually decreases over time. Its levels correlate with burn severity, highlighting its potential as a biomarker for injury assessment [71].
Abdominal injury	- Circulating 20S proteasome activity in blunt abdominal trauma patients peaks at 12–16 h post-injury before declining at 2–6 and 48 h, indicating a time-dependent pattern with a delayed peak response to tissue damage [72].
Tissue injury	- Circulating 20S proteasome is likely released from damaged tissues in response to injury and serves as a biomarker of tissue damage, with higher levels in burn patients compared to those with mild head injury or blunt abdominal trauma, suggesting its potential as a marker of immune activity and cellular degradation in trauma patients [72].
Inflammation-associated injury	- UCHL1 is expressed in podocytes and that its expression is significantly upregulated in acute and immune-mediated glomerular diseases such as acute proliferative glomerulonephritis (APGN), lupus nephritis (LN), membranous glomerulonephritis (MGN) and IgA nephropathy. In contrast, its expression is lower in in focal segmental glomerulosclerosis (FSGS), minimal change disease (MCD), minor abnormalities, and normal kidney tissues [74]. - UCHL1 and NF-κB are upregulated in immune complex-mediated glomerulonephritis, correlating with podocyte injury. This upregulation is absent in non-immune complex cases. In vitro, immune stimulation induces UCHL1 via the NF-κB pathway, linking immune activation to podocyte damage [75]. - UCHL1 is a potential biomarker for pancreatic beta cell injury in rats, showing strong beta cell-selectivity and consistent release upon cell damage [77]. - UCHL1 deficiency leads to protein accumulation, cellular stress, and increased susceptibility to renal injury, particularly in podocytes and endothelial cells, resulting in proteinuria, severe kidney damage, and acute renal failure [76].
Ischemia-reperfusion injury	- Proline-arginine-rich PR-39 peptide and its truncated form PR-11 effectively reduce myocardial ischemia-reperfusion injury by inhibiting proteasome-mediated IκBα degradation and subsequent NF-κB-dependent adhesion molecule expression. This leads to decreased infarct size, improved cardiac function, and reduced inflammation [84]. - Institute Georges Lopez-1 (IGL-1) solution more effectively prevents proteolysis, ATP breakdown, and ubiquitin proteasome system (UPS) activation during cold ischemia-reperfusion compared to University of Wisconsin (UW) solution. This protection reduces liver injury, mitochondrial damage, inflammation, and apoptosis, highlighting the role of proteasome inhibition in improving liver graft preservation [80].

## 5. Possible Clinical Implications of Proteasome Inhibition

Apoptosis, or programmed cell death, is essential for physiology, and the proteasomal system of protein degradation plays a key role in its regulation. The proteasome influences apoptosis by modulating levels of proapoptotic factors (such as p53, Bax, and NOXA) and antiapoptotic proteins (such as Bcl-2 and IAP). Inhibition of the proteasome increases proapoptotic factors and decreases antiapoptotic proteins, promoting apoptosis [86]. Several authors observed that inhibitors of the proteasome induce apoptosis in leukemic cells [87,88]. Experiments with proteasome inhibitors showed antiproliferative and proapoptotic activity against hematological and solid tumors [89]. Proteasome inhibition curbs the inflammation-associated transcription factor NFκB through stabilization of its inhibitor IκB [90]. The I kappa B kinase complex is a major regulator of the NF-κB signaling pathway. It consists of two catalytic kinase subunits, CHUK (IKBKA, IKK1, or IKKα) and IKBKB (IKK2 or IKKβ), as well as a regulatory non-catalytic subunit, IKBKG (NEMO—NF-κB essential modulator, IKK3, or IKKγ). Through phosphorylation, ubiquitination, and degradation of the IκB inhibitor, this complex leads to the release of NF-κB, which functions as a transcription factor for numerous genes within the cell nucleus. NFκB takes part in the regulation of immune and inflammatory responses and induces angiogenesis, proliferation, migration, and suppression of apoptosis during tumourogenesis [89,91,92,93,94].

Bortezomib, one of the proteasome inhibitors, has found application in the treatment of multiple myeloma. Bortezomib induces apoptosis in neoplastic cells and enhances their sensitivity to other chemotherapeutic agents. However, bortezomib treatment is associated with severe side effects and the development of drug resistance [94]. Henniger et al. demonstrated that bortezomib provided significant neuroprotection in animal models of cerebral ischemia and reperfusion, improving neurological outcomes without causing additional damage [95].

These observations prompted the search for new therapeutic targets. A new proteasome inhibitor, BSc2118, has shown anti-myeloma activity in animal models and has been used as a protective agent against cerebral ischemia in mice, inducing long-term neuroprotection through HIF1A accumulation and enhanced angioneurogenesis, with no observed side effects [96,97]. Additionally, proteasome inhibitors have demonstrated proapoptotic, antiproliferative, and anti-inflammatory effects on cardiovascular cells [98].

Chen et al. proved that inhibition of the immunoproteasome can reduce infarct volume and attenuate inflammatory reactions in animal models of ischemic stroke [98]. Furthermore, proteasome inhibitors have been shown to decrease liver damage after ischemia-reperfusion, with potential benefits for liver transplant patients [99]. However, contrary to previous reports, some studies have indicated that proteasome inhibition can hamper cell apoptosis in certain conditions, such as hypoxia-reoxygenation-related lung injury [100].

Moreover, proteasome blockers like lactacystin and β-lactone have been observed to prevent muscle proteolysis induced by thermal injury, potentially reducing the risk of infection and improving wound healing [101]. Despite these benefits, proteasome inhibition has also been associated with increased burn-associated mortality [102]. During the aging process, decreased proteasome activity leads to the accumulation of defective proteins, which are toxic to cells [103]. Some researchers are exploring methods to reverse the aging process through the activation of the 26S proteasome. Additionally, UCHL1 deletion has been shown to ameliorate glomerular injury in animal models of focal segmental glomerulosclerosis [104].

There is a clinical need for specific proteasome inhibitors targeting the 20S and 26S proteasomes due to their distinct roles in cellular processes and disease mechanisms. The 20S proteasome inhibitors, which target protein aggregation, may help manage neurodegenerative diseases and have potential in reducing inflammation and modulating immune responses. In contrast, the 26S proteasome inhibitors are used in treating hematologic malignancies like multiple myeloma and mantle cell lymphoma, disrupting the degradation of tumor suppressor proteins and leading to cancer cell death. Proteasome inhibitors like bortezomib have shown significant efficacy in treating multiple myeloma, and new inhibitors are being developed to reduce side effects and overcome drug resistance [105,106].

## 6. Conclusions

It is important to highlight the distinct and crucial roles of UCHL1, 26S, and 20S proteasome subunits as biomarkers. UCHL1 appears to be particularly relevant for identifying brain and neuronal damage and in advancing the diagnosis and prognosis of traumatic brain injury (TBI) and other neurological conditions. Meanwhile, the 26S and 20S proteasomes may serve as markers for peripheral tissue damage. This differentiation enhances our understanding and ability to target specific types of tissue damage in clinical settings. Based on the literature, specific proteasome inhibitors offer new opportunities for targeted therapies, improved patient outcomes, and minimized side effects in various clinical applications. These inhibitors hold promise not only in oncology but also in neurology and immunology.

## Data Availability

The data that support the findings of this study are available from the corresponding author upon reasonable request.

## Abbreviations

APGN	acute proliferative glomerulonephritis
Bcl-2	B-cell lymphoma 2 regulatory protein
Bax	proapoptotic protein
CNS	central nervous system
COX2	cyclooxygenase
c-proteasome	circulating proteasome
CT	computed tomography
DUB	deubiquitinating enzymes
FSGS	focal segmental glomerulosclerosis
IAP	inhibitor of apoptosis
IkB	Inhibitor of κB
IFNγ	interferon gamma
GFAP	glial fibrillary acidic protein
HECT	homologous to the E6AP carboxyl terminus
HIE	hypoxic-ischemic encephalopathy
HIF1A	hypoxia-inducible factor 1-alpha
IκB	inhibitor of nuclear factor kappa B
JAMM	Jab1/Mov34/Mpr1 metalloenzyme
MAP-2	microtubule-associated protein 2
MBP	myelin basic protein
MGN	membranous glomerulonephritis
MRI	magnetic resonance imaging
LN	lupus nephritis
NF-kβ	nuclear factor kβ
NOXA	proapoptotic protein
NSE	Neuron-Specific Enolase
NF-L	neurofilament Light
NSI	neurological severity index
OUT	ovarian tumor-like proteases
p53	tumor suppressor p53
PROTAC	proteolysis-targeting chimera
RING	really interesting new gene
SCI	spinal cord injury
TBI	traumatic brain injury
TNF-α	tumor necrosis factor
TBI	traumatic brain injury
Ub	ubiquitin
UCH	ubiquitin C-terminal hydrolases
UCHL1	ubiquitin carboxy-terminal hydrolase L1
UPP	ubiquitin-proteasome pathway
UPS	ubiquitin-proteasome system
USP	ubiquitin-specific proteases
